# Impact of mHealth-Augmented Social Support on Health Care Use Among Patients With Diabetes: Secondary Analysis of the TExT-MED+FANS Trial

**DOI:** 10.2196/65113

**Published:** 2026-02-27

**Authors:** Danielle Hazime, Liza Raffi, Elizabeth Burner

**Affiliations:** 1 Rush Medical College of Rush University Medical Center, Chicago, IL, United States; 2 Cancer Research Center for Health Equity, Cedars-Sinai Medical Center, 700 N San Vicente Boulevard, Suite G-500, Los Angeles, CA, 90069, United States, 1 3102185545; 3 Sol Price School of Public Policy, University of Southern California, Los Angeles, CA, United States; 4 Department of Internal Medicine, Cedars-Sinai Medical Center, Los Angeles, CA, United States; 5 Department of Emergency Medicine, Keck School of Medicine of USC, Los Angeles, CA, United States

**Keywords:** diabetes, health care use, mHealth, mobile health, social support, health care utilization

## Abstract

**Background:**

The rising cost of unscheduled acute health care, particularly for emergency department (ED) visits, poses significant financial burdens. In 2021, aggregate costs for treat-and-release ED visits in the United States accounted for an estimated US $80 billion, while the total annual cost of diabetes was US $412.9 billion in 2022—representing about 1 in every 4 health care dollars, 61% of which are directly linked to diabetes.

**Objective:**

This study explores the impact of a mobile health (mHealth) intervention with augmented social support delivered via guided SMS text messaging on health care use among patients with diabetes through a secondary analysis of the TExT-MED+FANS (Trial to Examine Text-Messaging in Emergency Patients With Diabetes + Family and Friends Network Support) randomized controlled trial.

**Methods:**

The trial involved 173 participants randomized into either a FANS mHealth-augmented social support or an active control group that received the same support curriculum via mailed pamphlet; “augmented” social support refers to recruiting both individuals with diabetes and a designated family member or friend (“supporter”) to participate. Supporters in the FANS arm received structured SMS guidance on how to assist the participant with diabetes in managing their condition. Health care use outcomes, including ED visits, hospitalizations, and clinic visits, were compared between groups during and after the intervention period using linear regression models on change in health care visits in the last year, with subgroup analysis by participant sex and supporter relationship.

**Results:**

Results showed significant reductions in acute unscheduled care visits for both groups during and after the intervention, with the FANS group experiencing a reduction of 1.04 visits during the intervention and 1.10 visits after the intervention, while the mailed pamphlet group had reductions of 1.47 and 1.53 visits, respectively (both *P*<.001). Clinic visits increased by 1.78 during the intervention phase (*P*=.01) but were not sustained postintervention. Hospitalizations modestly decreased in both groups, but the decrease was statistically significant only for the mailed pamphlet group (*P*=.002). Sex and supporter relationship differences were observed, with females supported by spouses in the active control group showing the largest decrease in unscheduled care visits.

**Conclusions:**

The findings suggest that mHealth interventions combined with structured social support can improve diabetes management and reduce health care costs. Although these conclusions are specific to this study, they align with prior research demonstrating the benefits of social support and mHealth interventions. These findings may inform future programs, including the design of low-cost, scalable interventions in resource-limited settings.

## Introduction

The cost of unscheduled, acute health care, particularly for emergency department (ED) care, continues to rise, with the strain particularly evident among patients with diabetes. In 2021, aggregate costs for treat-and-release ED visits in the United States accounted for an estimated US $80 billion in costs, with the total annual cost of diabetes being US $412.9 billion in 2022, representing about 1 in every 4 health care dollars—61% of which are directly linked to diabetes [1,2]. Patients with diabetes may need frequent unscheduled care due to complicated care needs, difficulty accessing care, and lack of self-management skills that result in clinical decompensation [3,4]. The US health care system often faces heavy financial burdens resulting from unscheduled visits [2,5]. In addition, high medical bills and unpredicted lost productivity ultimately pose significant expenses to patients themselves [6]. This is compounded by the broader societal impact, where increased health care spending can affect economic stability. Finding strategies to minimize these costs is important to creating a more sustainable health care ecosystem.

Within the context of such challenges, mobile health (mHealth) interventions offer an approach to managing chronic diseases like diabetes, providing tools such as glucose monitoring, medication reminders, dietary tracking, and personalized coaching [7,8]. These platforms aim to reduce health care costs, improve accessibility, and enhance patient medication taking, engagement, and involvement [9-11]. Examining the impact of mHealth interventions on health care use is crucial for evaluating their feasibility and scalability across various health care settings and populations, as they can greatly reduce direct costs [12,13].

Social support plays an important role in diabetes management, with prior studies showing that family and friend involvement can improve medication taking, self-management behaviors, and glycemic stability [14-16]. Recent work has also examined mHealth-directed social support, suggesting that combining digital health tools with structured support may further enhance patient engagement and outcomes [16]. However, few studies have directly compared mHealth-augmented support with traditional in-person support or print-based approaches. Such comparisons are essential to inform comprehensive diabetes management strategies that effectively leverage both technology and social networks to improve patient care and reduce costs associated with unscheduled health care use.

To understand the impact of mHealth integrated social support on health care use, this study offers a detailed analysis of health care use trends as a secondary analysis of TExT-MEDS+FANS (Trial to Examine Text-Messaging in Emergency Patients With Diabetes + Family and Friends Network Support), a randomized controlled trial of mHealth-augmented social support (FANS) compared to standard social support (mailed pamphlet) added to a patient-focused mHealth curriculum for patients with diabetes. The primary research objectives were to assess the association between mHealth-delivered social support and its influence on health care–seeking behaviors, including the frequency and nature of health care use. We also examined whether the character of the existing support associations prior to the intervention modified the intervention effect.

## Methods

### Research Methodology

TExT-MED+FANS was a 12-month comparative effectiveness randomized controlled trial that investigated the impact of two exposures: (1) mHealth-augmented social support (FANS) and (2) standard social support (mailed pamphlet) on patient health care use outcomes. Participants were enrolled from June 2017 to December 2019. This study represents a secondary analysis of the original randomized trial data, focused on differences in health care periods and subgroup characteristics not examined in the primary publication. For further information on the methods used in TExT-MED+FANS and the specific mHealth program under study, please refer to the protocol study by Burner et al [17].

### Ethical Considerations

The study received approval from the University of Southern California Health Sciences Institutional Review Board (HS-17‐00406). All participants provided written informed consent. Data were stored in an encrypted dataset and were not deidentified to allow linkage to electronic medical record (EMR) information. Consent procedures were conducted in English or Spanish, depending on patient preference. Patients and supporters were provided with compensation ranging from US $20 to US $280, depending on the number of study visits completed [16].

### Study Population

#### Study Protocol: Screening

Patients were eligible to participate if they had either type 1 or type 2 diabetes with elevated glycated hemoglobin (A_1c_) levels (A_1c_ ≥8.5), were aged 18 years or older, possessed the capability to send and receive text messages, could identify a supporter, and were able to provide informed consent.

#### Enrollment of Patient

Trained research assistants used convenience sampling by approaching eligible patients with diagnosed diabetes identified through their EMR during routine ED visits, regardless of the nature of the patient’s visit. No public advertisement or broader outreach strategy was used. Patients provided informed consent and completed an intake interview in their preferred language (English or Spanish). They were then registered in the SMS text-messaging platform to access the TExT-MED patient curriculum. While in the ED, patients nominated a family member or close friend to serve as their supporter for the study, and research assistants collected contact information for up to 3 supporters.

#### Enrollment of Support Person

Support person enrollment took place concurrently in the ED or through telephone communication within a 2-week window following patient enrollment. Supporters completed the enrollment process by providing informed consent and verifying their ability to send and receive text messages.

#### Randomization

Following supporter enrollment, patient-supporter dyads were randomized to either mHealth-augmented social support through the FANS program or standard social support delivered via mailed pamphlets. If the dyad was randomized to the FANS arm, research assistants registered the family member in the SMS text-messaging platform to receive the mHealth-augmented support curriculum. A computer-generated simple randomization sequence was used, with allocation concealed via sealed envelopes until assignment.

#### Treatment Groups: Exposures

The main exposure variable was the type of social support intervention: mHealth-augmented support (FANS) versus standard support (mailed pamphlet). Every patient received the SMS text message–based patient curriculum (TExT-MED) regardless of their group assignment. This included automated, static (noninteractive) text messages delivered daily for 6 months. The content included education on diabetes self-management, encouragement, and reminders. Messages were not personalized. Supporters in the intervention group received a text message–based curriculum (FANS) guiding them to provide mHealth-augmented social support to their loved ones. These messages were also static and noninteractive, delivered twice weekly. Supporters in the active control group received standard social support through the same material and curriculum distributed via a mailed pamphlet. No additional support or follow-up beyond the pamphlet was provided to the control group supporters.

### Measures: Outcomes and Assessments

The primary outcome was health care use, assessed through EMR review. Outcome variables that were included are given below:

1. Acute unscheduled care, defined as any visit to the Medical Center ED or urgent care.
2. Hospitalizations, defined as any inpatient stay recorded in the Los Angeles County Department of Health Services system.
3. Scheduled clinic visits, defined as fulfilled clinic appointments with a primary care provider within the Los Angeles County Department of Health Services.

All calculations underwent scrutiny by 2 reviewers, with any discrepancies resolved through consensus.

### Measurement Phases

The following measurement phases were used to assess changes in health care use over time:

- Prior: 6 months prior to enrollment.
- Active: from the intervention start date to the intervention end date (a 6-month period).
- After intervention: end of intervention date to 6 months after intervention. Participants did not receive SMS text messages during this period.

At the time of enrollment, patients self-reported their sex, race or ethnicity, primary language, duration of diabetes, and access to primary care. Age and insurance status were extracted from the EMR. Insurance status was classified as means-tested (eg, Medicaid or other government insurance programs), non–means-tested (eg, Medicare or private insurance), or uninsured (no recorded insurance coverage at the time of the visit). The supporter’s sex was reported by the supporter, and for analysis purposes, supporters identified as cohabitating significant others by the patient were considered spouses. Supporters other than spouses were categorized as nonspouse supporters, encompassing various relationships such as siblings, friends, or children.

### Data Analysis

The intervention group assignment was otherwise examined with an intention-to-treat approach, regardless of patient or supporter dropping out of the TExT-MED or FANS text messages. Analysis was completed with STATA (version 17.0; StataCorp LLC). Data were examined with histograms and did not violate assumptions of heteroscedasticity. Baseline characteristics were examined descriptively as a whole and by intervention group (Table 1).

In our main effects analysis, we used linear regression models with outcomes of change scores of health care visits from (1) baseline to the end of intervention phases, and (2) from the end of intervention to the end of maintenance phase, examining differences in health care use by the FANS augmented versus traditional mailed pamphlet groups (Table 2). The main independent variable was intervention group assignment. No other independent variables were included in the main effects model. Differences between groups were estimated with the contrast and margins function. We individually examined unscheduled acute care visits (ED and urgent care visits), hospitalizations, and scheduled care (clinic) visits. For subgroup analysis, we used an interaction term for sex and supporter relationship to examine the moderating effect of supporter relationship and sex with the same procedures (Table 3).

**Table 1.** Demographic data (N=165).

Characteristics	Active control (n=86)	FANS^a^ (n=79)	Combined
Age (y), mean (SD)	48.25 (11)	46.88 (10)	47.59 (10.5)
Sex (male), n (%)	37 (43)	45 (57)	82 (49.7)
Spouse as supporter, n (%)	37 (43)	29 (37)	66 (40)
Race or ethnicity, n (%)			
Asian	2 (2)	0 (0)	2 (1.2)
Black	3 (3)	6 (8)	9 (5.5)
Hispanic	81 (94)	71 (90)	152 (92.1)
Non-Hispanic White	0 (0)	2 (3)	2 (1.2)
Primary language (Spanish), n (%)	64 (74)	51 (65)	115 (69.7)
Insurance type, n (%)			
Means-based	82 (95)	71 (90)	153 (92.7)
Non–means-based	2 (2)	5 (6)	7 (4.2)
Uninsured	2 (2)	3 (4)	5 (3)
Self-reported at least 1 primary care visit(s) in 6 months prior to study, n (%)	64 (74)	63 (80)	127 (77)
Years with diabetes, mean (SD)	11.65 (9)	11.10 (9)	11.39 (8.7)
Did not complete full curriculum, n (%)	6 (7)	4 (5)	10 (6.1)

a FANS: Family and Friends Network Support.

**Table 2.** Main outcomes (N=165)^a^.

Outcome	Active control (mailed pamphlet; n=86)		FANS^b^ (mHealth-augmented social support; n=79)		Between-group difference in change score (active control—FANS)		Combined total study population	
	Mean (95% CI)	*P* value	Mean (95% CI)	*P* value	Mean (95% CI)	*P* value	Mean (95% CI)	*P* value
$\overset{-}{∆}$Acute unscheduled care visits intervention phase versus patient’s baseline	−1.47 (−1.87 to −1.06)	<.001	−1.04 (−1.41 to −0.66)	<.001	−0.43 (−0.98 to 0.12)	.13	−1.26 (−1.54 to −0.99)	<.001
$\overset{-}{∆}$Acute unscheduled care visits postintervention phase versus patient’s baseline	−1.53 (−1.93 to −1.14)	<.001	−1.10 (−1.52 to −0.69)	<.001	−0.43 (−1.00 to 0.13)	.13	−1.33 (−1.61 to −1.04)	<.001
$\overset{-}{∆}$Clinic visits intervention phase versus patient’s baseline	1.00 (0.13 to 1.80)	.02	1.78 (0.40 to 3.17)	.01	−0.82 (−2.39 to 0.75)	.31	1.36 (0.57 to 2.14)	<.001
$\overset{-}{∆}$Clinic visits postintervention phase versus patient’s baseline	−0.14 (−1.20 to 0.92)	.79	0.48 (−0.81 to 1.77)	.46	−0.62 (−2.26 to 1.02)	.46	0.16 (0.81 to 1.00)	.70
$\overset{-}{∆}$Hospitalizations (intervention phase vs patient’s baseline)	−0.24 (−0.40 to 00.09)	.002	−0.18 (−0.37 to 0.01)	.07	−0.07 (−0.31 to 0.17)	.58	−0.21 (−0.33 to −0.09)	<.001
$\overset{-}{∆}$Hospitalizations (postintervention phase vs patient’s baseline	−0.22 (−0.38 to −0.06)	.01	−0.20 (−0.44 to 0.04)	.01	−0.02 (−0.30 to 0.26)	.90	−0.21 (−0.35 to −0.07)	.003

a Values represent the mean change in health care use (with 95% CIs) from baseline to each study period for the intervention and control groups. *P* values reflect the significance of within-group changes, while the between-group difference column represents the difference in change scores between groups.

b FANS: Family and Friends Network Support.

**Table 3.** Sex and supporter type subanalysis^a^.

	Female with spouse supporters, mean (95% CI; *P* value)		Females with nonspouse supporters, mean (95% CI; *P* value)		Male with spouse supporters, mean (95% CI; *P* value)		Males with nonspouse supporters, mean (95% CI; *P* value)	
	Active control	FANS^b^	Active control	FANS	Active control	FANS	Active control	FANS
Acute unscheduled care								
$\overset{-}{∆}$6-month	−2.41 (−3.24 to −1.59*; P*<.001)	0.63 (0.58 to 1.83*; P*=.31)	−1.25 (−1.85 to −0.65*; P*<.001)	–1.58 (-2.25 to -0.91*; P*<.001)	–1.05 (-1.81 to -0.29*; P*=.007)	–1.19 (-1.93 to -0.45*; P*=.002)	–1.41 (–2.24 to –0.59*; P*<.001)	–0.88 (–1.57 to –0.18*; P=*.01)
$\overset{-}{∆}$12-month	−2.76 (−3.61 to −1.92*; P*<.001)	0.38 (−0.86 to 1.61*; P*=0.55)	−1.25 (−1.87 to −0.63*; P*<.001)	−1.42 (−2.11 to −0.74*; P*<.001)	−0.9 (−1.68 to −0.12*; P*=.02)	−1.00 (−1.76 to −0.24*; P*=0.01)	−1.59 (–2.44 to –0.74*; P*<.001)	–1.33 (–2.05 to –0.62*; P*<.001)
Clinic visits								
$\overset{-}{∆}$6-month	2.47 (0.05 to 4.89*; P*=.05)	4.00 (0.47 to 7.53) (*P*=.03)	1.50 (−0.26 to 3.3) (*P=.10)*	0.04 (−1.92 to 1.99) (*P*=.97)	−0.35 (−2.58 to 1.88) (*P*=.76)	1.48 (−0.70 to 3.65) (*P*=.18)	0.00 (−2.42 to 2.42) (*P*=1.00)	3.21 (1.17 to 5.24) (*P*=.002*)*
$\overset{-}{∆}$12-month	1.12 (−1.40 to 3.63*; P*=.38)	4.75 (1.08 to 8.42*; P*=.011)	0.03 (−1.80 to 1.87*; P*=.97)	−0.62 (−2.65 to 1.42*; P*=.55)	−1.55 (−3.87 to 0.77*; P*=.19)	−1.05 (−3.31 to 1.22*; P*=.36)	−0.06 (−2.57 to 2.46*; P*=.96)	1.58 (−0.53 to 3.70*; P*=.14)
Hospitalizations								
$\overset{-}{∆}$6-month	−0.18 (−0.55 to 0.20*; P*=.36)	−0.38 (−0.92 to 0.17*; P*=.18)	−0.28 (−0.56 to −0.01*; P*=.05)	0.04 (−0.27 to 0.34*; P*=.80)	−0.25 (−0.60 to 0.10*; P*=.16)	−0.24 (−0.58 to 0.10*; P*=.17)	−0.24 (−0.61 to 0.14*; P*=.22)	−0.29 (−0.61 to 0.03*; P*=.07)
$\overset{-}{∆}$12-month	−0.24 (−0.68 to 0.21*; P*=.30)	-0.25 (−0.90 to 0.40*; P*=.45)	−0.19 (−0.51 to 0.14*; P*=.26)	−0.12 (−0.48 to 0.24*; P*=.53)	−0.3 (−0.71 to 0.11*; P*=.15)	−0.05 (−0.45 to 0.35*; P*=.82)	−0.18 (−0.62 to 0.27*; P*=.44)	−0.42 (−0.79 to −0.04*; P*=.03)

a Values represent the mean change in health care use (with 95% CIs) by sex and supporter relationship for each study period. *P* values indicate the significance of within-group changes.

b FANS: Family and Friends Network Support.

## Results

**Figure 1.**
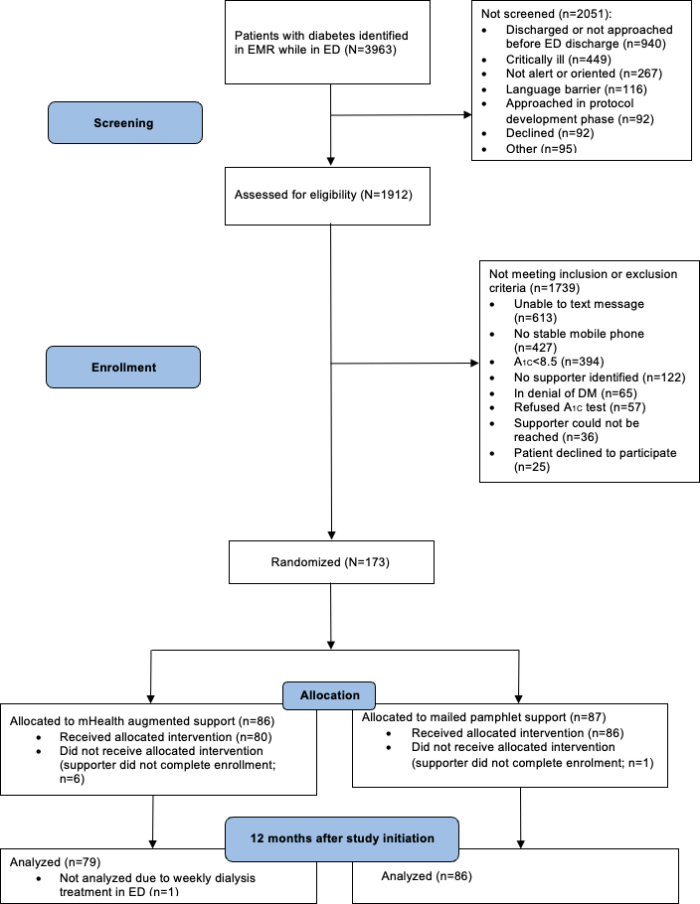
Participant flow diagram. DM: diabetes mellitus; ED: emergency department; EMR: electronic medical record; FANS: Family and Friends Network Support; mHealth: mobile health.

In this study, a total of 3963 patients with diabetes were identified from electronic health records while in the ED (Figure 1). Of these, 12051 patients were not screened for various reasons, including critical illness, discharge or nonapproach before ED discharge, lack of alertness and orientation, language barriers, and other reasons. Among the screened individuals, 1739 were excluded due to inability to send SMS text messages, lack of a stable mobile phone, A_1c_ <8.5%, absence of a supportive individual, and other reasons (n=183; Figure 1). Ultimately, 173 patients were randomized into the study and allocated either to mHealth-augmented support (86/173, 49.7%) or standard social support (mailed pamphlet; 87/173, 50.3%). One participant in the mHealth-augmented group was excluded from this analysis because they were undergoing thrice-weekly dialysis treatment in the ED due to insurance limitations during the study period. The final analytic population was composed of 165 participants randomized into 2 intervention groups: FANS with 79 participants and a mailed pamphlet with 86 participants.

The average age of a patient was 47.59 (10.5) years, and males comprised approximately half of the study population, with a slightly higher proportion in the FANS group (45/79, 57%) compared to the mailed pamphlet group (37/86, 43%). The majority of participants self-identified as Latino or Hispanic (152/165, 92.1%), with small representations from Non-Latino or Hispanic White (2/165, 1.2%), Asian (2/165, 1.2%), and Black (9/165, 5.5%) backgrounds. A substantial proportion of participants primarily spoke Spanish (115/165, 69.7%). Insurance coverage was predominantly means-based (Medicaid or other means-based government insurance programs; 153/165, 92.7%), with a few participants either non–means-based insured (Medicare or private insurance) (7/165, 4.2%) or uninsured (5/165, 3%). Most participants had visited a primary care provider within the previous 6 months (127/165, 77%). The average duration of diabetes among the participants was 11.39 (9.7) years, indicating a long-standing condition of diabetes within the cohort (Table 1).

The results from the study indicated significant changes in health care use across different types of care visits during the intervention and postintervention phases (Table 2). During the intervention phase, there was a notable reduction in acute unscheduled care visits across both intervention groups. The FANS group experienced an average decrease of 1.04 visits (*P*<.001), while the mailed pamphlet group saw a larger average decrease of 1.47 visits (*P*<.001). This trend was sustained after intervention, with reductions of 1.10 and 1.53 visits, respectively, for FANS and mailed pamphlet groups, both statistically significant (*P*<.001). The difference between the FANS and mailed pamphlet groups was not statistically significant in either period.

Clinic visits increased during the intervention phase, with the FANS group showing a mean increase of 1.78 visits (*P*=.01) and the mailed pamphlet group an increase of 1.00 visits (*P*=.02). However, this increase was not sustained after intervention, with the changes becoming statistically insignificant and the direction of change mixed. There was a difference of 0.62 visits between the intervention groups during the postintervention period; however, this was not statistically significant.

Hospitalization rates showed a modest decrease during the intervention phase with reductions of 0.24 visits in the mailed pamphlet group and 0.18 visits in the FANS group, with the former being statistically significant (*P*=.002). This decreasing trend in hospitalizations continued into the postintervention phase with similar reductions. There was a difference of −0.07 between the intervention groups, though this was not statistically significant.

During the 6-month interval, females with spouse supporters in the mailed pamphlet group experienced a significant decrease in acute unscheduled care visits, showing a reduction of 2.41 visits (*P*<.001). In contrast, males with spouse supporters saw a decrease of 1.19 visits (*P*=.002) in the FANS group and a similar decrease in the mailed pamphlet group. Females with nonspouse supporters and males with nonspouse supporters also showed significant decreases, though the effect was strongest in those with spouse supporters. Clinic visits increased significantly during the 6-month interval for both females and males with spouse supporters in the FANS group, with increases of 4 visits (*P*=.03) and 3.21 visits (*P*=.002), respectively.

## Discussion

### Principal Findings

In this study, we evaluated the impact of a mHealth-augmented social support intervention (FANS) compared to an active control (mailed pamphlet) on health care use among patients with diabetes, analyzing data from 165 participants to assess changes in acute unscheduled care visits, clinic visits, and hospitalizations. The main findings indicate a significant reduction in acute unscheduled care visits and hospitalizations for both intervention and active control groups, with this effect maintained after intervention. Moreover, an increase in clinic visits was seen during both the intervention and postintervention phases. In addition, we found that the type of supporter, particularly whether or not the supporter was a spouse, played a role in study participants’ use of the mentioned health services.

The reduction in acute unscheduled care visits aligns with existing literature supporting the effectiveness of mHealth interventions and educational pamphlets in improving patient self-management [18]. Previous studies have shown that providing structured support and education to patients with chronic diseases can lead to better health outcomes [19]. Specifically for patients with diabetes, these interventions have been linked to improved diabetes care, glycemic stability, and reduced A_1c_ levels [20]. Moreover, the integration of mHealth technologies has facilitated more consistent monitoring and timely interventions, enhancing overall disease management. Such approaches not only empower patients but also foster a proactive engagement in their health care journey, ultimately reducing the burden on acute care services [21].

There is a notable gap in the literature regarding health care use following mHealth interventions, particularly concerning ED visits, hospitalizations, and clinic visits for patients with diabetes. While extensive data exists on the impact of such interventions on other chronic conditions, like chronic obstructive pulmonary disease (COPD), similar comprehensive studies on diabetes-focused mHealth interventions are lacking. Yang et al [22] demonstrated that an mHealth curriculum for patients with COPD significantly reduced the risk of hospitalizations, although it did not yield a substantial difference in the average length of stay. In this study focusing on diabetes, a marked reduction in the frequency of hospitalizations was observed both during and after the intervention; however, we did not examine the length of stay. While both conditions benefit from mHealth interventions, the nature and progression of diabetes and COPD may present unique challenges and opportunities for reducing healthcare utilization, emphasizing the need for more tailored research in diabetes-specific programs.

The subgroup analysis revealed that while acute unscheduled care visits decreased significantly across most supporter groups, females with spouse supporters in the FANS group showed no significant change. The type of supporter may influence the intervention’s effectiveness, aligning with prior work indicating that spousal support by sex can vary widely in its impact on health behaviors [23]. These patterns underscore the complex role of social support dynamics, as highlighted in previous studies which have shown that the quality and type of support can differentially impact health outcomes [24,25]. Specifically, research has emphasized the effectiveness of spousal support in chronic disease management, including diabetes, due to the consistent and close nature of the support provided [26]. Clinic visits increased significantly for females with spouse supporters during the intervention phase in both groups, which aligns with studies indicating that females are generally more proactive in seeking preventive care [27-29]. In this study, this increase was only sustained in the FANS group, suggesting the potential for digital health tools to maintain engagement with health care services. This study’s findings align with these insights, suggesting that while mHealth interventions can be broadly beneficial, their impact may be moderated by the type of support relationship involved.

The findings of this study have substantial implications for public health and policy. The reduction in acute unscheduled care visits among patients with diabetes through mHealth-augmented social support interventions highlights the potential of such technologies in enhancing chronic disease management. Policy makers should consider integrating mHealth solutions into standard care protocols, particularly for populations with limited access to care, to improve health care accessibility and outcomes [20,30,31]. In addition, investing in mHealth platforms could alleviate the financial burden on health care systems by reducing the frequency of costly acute unscheduled care visits and hospitalizations [32,33]. Public health initiatives should also focus on promoting digital literacy and access to mHealth technologies, ensuring that communities that have been historically marginalized can fully benefit from these advancements. By incorporating mHealth interventions into broader health care strategies, a more sustainable and equitable health care system can be created that better addresses the needs of patients with chronic conditions like diabetes. Future research and policy initiatives should also consider incorporating structured family or supporter-based engagement as a mechanism to enhance intervention effectiveness and long-term adherence.

This study has several limitations that must be considered when interpreting the findings. First, the small sample size and the secondary analysis nature of the study may limit the internal validity of the results. Using secondary data introduces inherent limitations, including potential bias related to how the original study was designed and executed. Since this analysis was not prespecified in the original study protocol, there may be risks of selective reporting or post hoc interpretation. In addition, certain variables of interest may not have been collected or controlled for in the original trial design, potentially limiting the scope of inferences. Future research with larger, more diverse populations is needed to confirm these findings. Second, acute unscheduled care visits in this study included both urgent care and ED visits, due to the structure of the Los Angeles County Department of Health Service Electronic Health Record System. While urgent care visits are less costly than ED visits, they still are inferior to scheduled clinic care and are not the desired health care use pattern [34,35]. Third, the sample was predominantly composed of individuals from Hispanic or Latino and low-socioeconomic backgrounds who used means-based insurance. These findings may not be generalizable to populations with different demographic and socioeconomic characteristics. Despite these limitations, the study provides valuable insights into the potential benefits of mHealth interventions. Addressing limitations and exploring tailored approaches can enhance the efficacy and reach of such interventions.

### Conclusions

This study contributes to the growing body of evidence examining the use of mHealth tools to support chronic disease management, reduce the burden on health care systems, and support the effectiveness of structured support systems and educational interventions in enhancing patient self-management and reducing health care use. mHealth-augmented social support interventions, such as the FANS program, may be able to reduce acute unscheduled care visits and hospitalizations among patients with diabetes. Though more work in this area is needed to identify who will most benefit, we saw indications that these interventions, particularly when involving spouse supporters, can lead to meaningful improvements in health care outcomes and engagement with preventive care services. The significant reductions in unscheduled care visits suggest that mHealth-augmented social support can effectively improve diabetes management, particularly for disproportionately affected groups. These findings underscore the importance of incorporating digital health solutions into chronic disease management social support strategies to improve health care outcomes and reduce acute care reliance.

## Abbreviations

A_1c_: glycated hemoglobin
COPD: chronic obstructive pulmonary disease
ED: emergency department
EMR: electronic medical record
FANS: Family and Friends Network Support
mHealth: mobile health
TExT-MED: trial to examine text-messaging in emergency patients with diabetes
